# Association between Upper-airway Surgery and Ameliorative Risk Markers of Endothelial Function in Obstructive Sleep Apnea

**DOI:** 10.1038/s41598-019-56601-w

**Published:** 2019-12-27

**Authors:** Fan Wang, Yuenan Liu, Huajun Xu, Yingjun Qian, Jianyin Zou, Hongliang Yi, Jian Guan, Shankai Yin

**Affiliations:** 1 Department of Otolaryngology Head and Neck Surgery & Center of Sleep Medicine, Shanghai Jiao Tong University Affiliated Sixth People’s Hospital, Yishan Road 600, Shanghai, 200233 China; 2 Shanghai Key Laboratory of Sleep Disordered Breathing, Yishan Road 600, Shanghai, 200233 China; 3 Clinical Research Center, Shanghai Jiao Tong University School of Medicine, South Chongqing Road 225, 200020 Shanghai, China

**Keywords:** Diagnostic markers, Predictive markers

## Abstract

The objective of our study was to evaluate the effects of upper-airway surgery on improvement of endothelial function-related markers in patients with obstructive sleep apnea (OSA). Subjects with moderate to severe OSA who underwent upper-airway surgery, with a follow-up duration of at least 6 months, were included. Pre- and postoperative polysomnographic variables and endothelial function-related markers were compared. Subgroup and correlation analyses were conducted to find possible indicators for better endothelial function-related markers after upper-airway surgery. In total, 44 patients with OSA were included. The mean follow-up duration was 1.72 ± 0.92 years. Serum VEGFA [−20.29 (CI: −35.27, −5.31), p < 0.05], Ang2 [−0.06 (CI: −0.16, 0.03), p < 0.05], E-selectin [−7.21 (CI: −11.01, −3.41), p < 0.001], VWF [−58.83 (CI: −103.93, −13.73), p < 0.05], VWFCP [−33.52 (CI: −66.34, −0.70), p < 0.05], and TM [−0.06 (CI: −0.09, −0.03), p < 0.05] were significantly lower after upper-airway surgery. However, other risk markers of endothelial function, such as Ang1, ICAM1, VEGFR1, and VCAM, did not change significantly. Correlations between improved endothelial function-related markers and ameliorated oxyhemoglobin saturation and glucolipid metabolism were established. Upper-airway surgery might be associated with an improvement in endothelial function in patients with OSA. These changes may be associated with improved oxygen saturation after upper-airway surgery.

## Introduction

Obstructive sleep apnea (OSA), one of most common types of sleep-disordered breathing, affects 24% of middle-aged men and 9% of women and has attracted widespread attention in recent years^1^. Mounting evidence suggests that the morbidity and mortality of untreated OSA are mostly attributable to cardiovascular complications^2,3^, primarily because of atherosclerosis-associated thrombosis and arterial stenosis. The vascular endothelial injury of OSA is reportedly associated with intermittent nocturnal hypoxia and sleep fragmentation-induced pathophysiological processes, including oxidative stress damage, inflammation, coagulation, and angiogenesis. Hence, inflammatory adhesion molecules, endothelial cell (EC) propermeability, inflammatory factor overflow, hypercoagulability, and downregulation of endothelium-stabilizing markers are thought to be pivotal biomarkers for endothelial injury. Previous studies reported higher circulating levels of VWF^4,5^ cell adhesion molecules (CAMs)^6–8^: intercellular adhesion molecule-1 (ICAM1), vascular cell adhesion molecule (VCAM), E-selectin in OSA patients compared with healthy individuals. Whether effective therapy of OSA could improve these endothelial injury biomarkers has become a focus of research and might be crucial in the interaction between OSA and cardiovascular complications.

Prospective studies have revealed that continuous positive airway pressure (CPAP) reduces cardiovascular risk in patients with OSA^2,9^. Additionally, a large observational study revealed that CPAP therapy reduced fatal cardiovascular death due to fatal myocardial infarction or stroke and nonfatal cardiovascular events such as non-fatal myocardial infarction, stroke, and acute coronary insufficiency in men with severe OSA compared with a population-based sample of age- and body mass index–matched healthy men^9^. There were also a large number of researches which reported ameliorative endotheliocyte dysfunction-related cytokines including VCAM^6^, ICAM1^7^, E-selectin^8^, von Willebrand factor (vWF)^10^ and other common inflammation markers with treatment of CPAP. Despite positive outcomes, a significant proportion of patients are unable to tolerate CPAP, which is a life-long treatment and may not change the structure of the upper airway substantially. Possible reasons for poor adherence include a self-reported oppressing sensation from the mask and continuous pressure, nasal symptoms, and psychological factors^9,11^.

Surgical procedures to expand the upper airway are an important alternative choice for OSA patients, especially those with surgically correctable obstructive anatomical defects. Previous research has revealed that upper-airway surgeries are associated with improved cardiometabolic biomarkers and even overall cardiovascular risk^12,13^. However, it is still not clear whether upper-airway surgeries aimed at solving oropharyngeal and retrolingual plane obstruction could also be associated with disorders of representative vascular biological function markers triggered by OSA-related pathological factors.

Therefore, we comprehensively analyzed the associations between upper-airway surgery and changes in EC dysfunction-related cytokines, including angiogenesis-related cytokines^14,15^: VEGFA, VEGF receptor 1 (VEGFR1), angiopoietin-1 (Ang1) and angiopoietin-2 (Ang2); CAMs^16^: ICAM-1, VCAM-1 and E-selectin; and coagulation function-related cytokines^17,18^: VWF, von Willebrand factor cleavage protease (VWFCP), and thrombomodulin (TM).

## Materials and Methods

### Study participants

**Figure 1:**
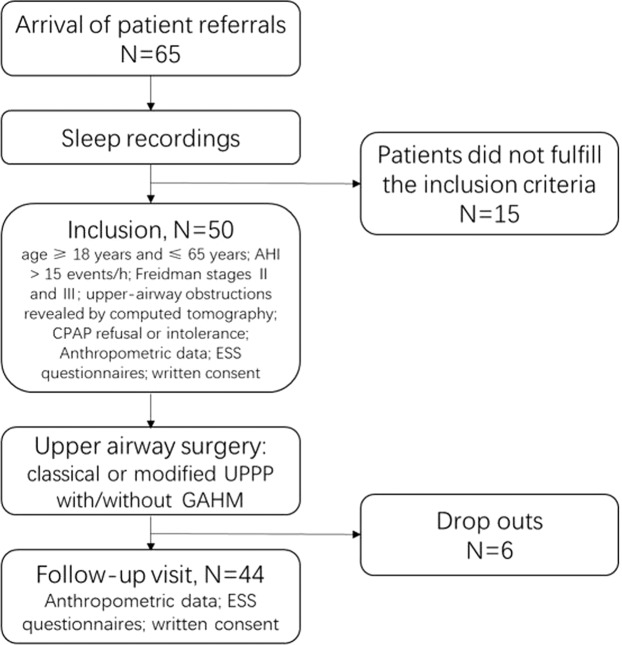
Flow chart of recruiting participants in our study.

This is a retrospective study and performed at a large teaching hospital (Shanghai Jiao Tong University (SJTU)-Affiliated Sixth People’s Hospital). The subjects in this study accepted upper airway surgery for palatopharyngeal plus/not plus glossopharyngeal obstructions, including classical or modified uvulopalatopharyngoplasty (UPPP) with or without genioglossus advancement and hyoid suspension (GAHM). All participants completed a follow-up visit at least 6 months after surgery in the Department of Otolaryngology and Head and Neck Surgery of the Shanghai Jiao Tong University Affiliated Sixth People’s Hospital between 2004 and 2013. All participants signed an informed consent form of their own accord. The inclusion criteria consisted of moderate or severe OSA [apnea/hypopnea index (AHI) > 15 events/h] determined by polysomnography (PSG); CPAP refusal or intolerance; age ≥ 18 years and ≤ 65 years; Freidman stages II and III; and upper-airway obstructions revealed by computed tomography. All patients underwent PSG examination during follow-up. The exclusion criteria included previous OSA treatment (including medications, oral appliances, surgeries, and CPAP) prior to our study; severe craniofacial maxillofacial malformation, obviously small tongue or macroglossia disease, or nasal cavity or nasopharynx obstruction; significant (>5%) postoperative change in body mass index (BMI); acute or chronic cardiorespiratory, hepatic, or nephric diseases; neurological disease and sleep disorders; acceptance of glucose- or lipid-lowering or anti-hypertension medications; and mental disease or psychological disorder. The flow chart of recruiting participants were presented in Figure 1. This study was conducted in accordance with the Declaration of Helsinki and was approved by the ethics committee of Shanghai Jiao Tong University Affiliated Sixth People’s Hospital.

### Sleep evaluation

Every participant underwent all-night standardized PSG monitoring (Alice 4 or 5; Respironics, Pittsburgh, PA, USA) at the sleep center of our clinical institute before and after surgery to objectively evaluate sleep condition. Nocturnal PSG included electroencephalography (EEG) (F3, F4, C3, C4, O3, O4 leads), electrooculography, chin and leg electromyography, nasal and oral airflow measures, thoracic abdominal movement measurement, oxygen saturation measurement, electrocardiography, snoring measurement, and body position. The PSG data were manually assessed by professional technicians in strict accordance with the 2007 criteria of the American Academy of Sleep Medicine (AASM)^19^. Obstructive apnea was defined as the absence of airflow for at least 10 seconds, or ≥90% decrease of airflow for at least 10 seconds. Hypopnea was defined by a greater than 50% reduction of airflow lasting ≥10 seconds accompanied by reduction of oxygen saturation ≥4% or occurrence of an arousal during sleep. The AHI was the number of apnea and hypopnea events per hour of sleep. OSA was diagnosed if the AHI was ≥5 [mild (5 ≤ AHI < 15), moderate (15 ≤ AHI < 30), or severe (AHI ≥ 30)]. Arousal was defined as abrupt switches in frequency of EEG ≥3 seconds. The micro-arousal index (MAI) was defined as the average number of microarousals per hour of total sleep time. The oxygen desaturation index (ODI) was the number of desaturations ≥4% per hour of total sleep time. The percentage of time with oxyhemoglobin saturation below 90% (CT90), mean saturation level (mean SaO_2_), and lowest saturation level (LSaO_2_) were also calculated. The Chinese version of the Epworth Sleepiness Scale (ESS) was applied to determine excessive daytime sleepiness or hypersomnia. Total score >10 was considered to indicate clinically significant hypersomnia.

### Surgical procedure

The operative technique was based on the preoperative evaluation of obstruction levels and patient preferences. Surgeries performed in those with palatal obstructions included UPPP, Han-UPPP, and Z-palatopharyngoplasty (ZPPP); in patients with multilevel obstructions, these procedures were combined with GAHM. All operations were finished by the same set of experienced doctors. Surgical success was defined as ≥50% reduction in the preoperative AHI and a postoperative AHI <20/h^20^.

### Clinical and biochemical measurement

Physical examinations, questionnaire surveys, and blood tests were performed twice, on the mornings after the preoperative and postoperative PSG monitoring; this included anthropometric indices covering height, weight, and waist circumference (WC). The means of measurements were calculated. Body mass index (BMI) was calculated as weight/height squared (kg/m^2^). Conforming to the recommendations of the Working Group on Obesity in China, obesity was defined as BMI ≥28 kg/m^2^, and abdominal obesity was defined as waist circumference ≥90 cm in males and ≥85 cm in females. Systolic blood pressure (SBP) and diastolic blood pressure (DBP) were measured to the nearest 2 mmHg by mercury sphygmomanometry, with all patients sitting after 15 minutes rest. High blood pressure was defined when SBP ≥140 mmHg or DBP ≥90 mmHg^21^. Age, sex, and medical history, including nature of the disease, timing of first diagnosis, and treatments, were also recorded. We applied and translated the Epworth sleepiness scale (ESS) questionnaire^22^ surveys into Chinese version to collect self-administered information referring to eight situations when OSA patients are known to be soporific based on their usual way of life.

After peripheral blood was drawn from each subject on the morning after PSG monitoring, blood samples were centrifuged at 3000 rpm for 10 min following standing at normal temperature for 30 min. The serum profile including glucose, insulin, triglycerides (TG), total cholesterol (TC), high-density lipoprotein cholesterol (HDL-C), low-density lipoprotein cholesterol (LDL-C), apolipoprotein A (Apo A), and apolipoprotein B (Apo B), was examined at the hospital’s clinical laboratory. Following the World Health Organization criteria^23^, hyperglycemia was diagnosed with fasting plasma glucose ≥7 mmol/L. The homeostatic model of insulin resistance (HOMA-IR) was calculated as glucose (mmol/L) × insulin (μU/mL)/22.5. Insulin resistance (IR) was defined as a HOMA-IR value >2.50^24^. Referring to the National Cholesterol Education Program Adult Treatment^25^, normal fasting triglyceride level was <1.7 mmol/L. High triglyceride (TG) was ≥2.3 mmol/L. Borderline-high triglyceride was ≥1.7 mmol/L and <2.3 mmol/L. Normal TC was diagnosed when the fasting total cholesterol level was <5.2 mmol/L. High TC was ≥6.2 mmol/L. Borderline high TC was ≥5.2 mmol/L and <6.2 mmol/L. Normal HDL-C was diagnosed when the fasting HDL-C was ≥1.03 mmol/L. High HDL-C was <1.03 mmol/L. Normal LDL-C was diagnosed when the fasting LDL-C level was <3.4 mmol/L. High LDL-C was ≥4.1 mmol/L. Borderline high LDL-C was ≥3.4 mmol/L and <4.1 mmol/L. Normal Apo A was diagnosed when the fasting level was ≥1.2 g/L and ≤16 g/L. Low Apo A was <1.2 g/L. Normal Apo B was ≥0.8 g/L and ≤1.1 g/L. High Apo B was >1.1 g/L.

The resting serum was stored at –80 °C for further endothelial injury biomarker analysis. Concentrations of serum VEGFA, VEGFR1, Ang1, Ang2, ICAM1, VCAM, E-selectin, VWF, VWFCP, and TM were measured using commercially available ELISA kits (Cloud-Clone Corp, Katy, TX, USA) in duplicate according to the manufacturer’s instructions. Each serum sample was loaded twice to guarantee the accuracy of detection.

### Statistical analysis

Study data were presented with descriptive statistics such as mean, median, standard deviation, 95% confidence interval (CI), and percentage. The normality of distribution of continuous variables was analyzed using the Kolmogorov-Smirnov test. The change between preoperative and postoperative variables with time was analyzed by paired sample t-test for normally distributed variables. Variables with skewed distributions were analyzed with the Wilcoxon signed rank test. We used Mann-Whitney or Kruskal-Wallis among-subgroup tests to evaluate the possible predictive utility of marker levels (VEGFA, VEGFR1, Ang1, Ang2, ICAM1, VCAM, E-selectin, VWF, VWFCP, and TM) by sex (male or female), preoperative OSA severity (AHI <30 or >30), preoperative obesity (BMI ≥28 kg/m^2^ or <28 kg/m^2^), preoperative abdominal obesity (yes or no), follow-up duration (<1 year or ≥1 year), successful operation or not, preoperative blood pressure levels, and preoperative metabolic status of blood glucose and lipids. Spearman’s rank correlation coefficients were calculated to determine the influence of PSG changes on the levels of variables that differed significantly pre- and postoperatively. Multivariable linear regression analyses with robust variances were performed to assess specific improved PSG parameters as a dependent variable to influence levels of EC markers. Statistical analyses were performed using SPSS software (Statistical Package for Social Sciences, version 22.0, SPSS Inc., Chicago, IL, USA). Two-sided P values < 0.05 were considered significant.

### Ethical approval

All procedures performed in studies involving human participants were in accordance with the ethical standards of the institutional research committee and with the 1964 Helsinki declaration and its later amendments.

### Informed consent

Informed consent was obtained from all individual participants included in the study.

## Results

### Subject characteristics

In total, 44 participants were included in this study. The male:female ratio was approximately 5:1. The preoperative ages were 22–60 years, and the average BMI was about 27.53 kg/m² (range 24.98–30.08). Subjects underwent UPPP or ZPPP with/without GAHM. The mean follow-up time was 1.72 ± 0.92 years, and the median follow-up time was 1.4 years. Table 1 lists the demographic characteristics of subjects.

**Table 1:** Baseline characteristics of patients undergoing surgery to treat OSA.

Variables	Preoperative
Number	44
Males (%)	37 (84)
Age (years)	41.27 ± 8.84
Height	1.71 ± 0.07
Weight	81.01 ± 10.51
WC (cm)	98.05 ± 7.50
BMI (Kg/m^2^)	27.53 ± 2.55
Successful surgery (%)	22 (50)
Follow-up duration (months)	24.33 ± 20.43
Abdominal obesity (%)	23(52)
Hypertention (%)	15(34)
Hyperglycaemia (%)	24(55)
IR (%)	2(5)
Diabetes	0
Smoker(%)	8(22)
Alcoholic(%)	3(8)

Abbreviations: WC = waist circumference, BMI = body mass index, IR = insulin resistance.

### Changes in sleep biomarkers and serum metabolic biomarkers

The preoperative AHIs were about 53.95/h (range from 49.65/h to 61.92/h). The proportion of surgical success was 40%. Postoperative sleep parameters, including AHI, ODI, CT90, MAI, ESS, mean SpO_2_, and lowest SpO_2_, all improved (p < 0.01). Table 2 lists the demographic characteristics and sleep data of subjects in the preoperative and postoperative groups.

**Table 2:** Comparisons of levels of glucolipid metabolism biomarkers and sleep parameters between preoperative and postoperative.

Variables	Preoperative	Postoperative	Mean Change (95% CI)	*p value*
**Anthropometric data**				
BMI (Kg/m^2^)	27.53 ± 2.55	27.95 ± 2.88	−0.68 (−1.13, −0.23)	0.376
WC (cm)	98.05 ± 7.50	98.10 ± 7.75	−0.64 (−2.24, 0.97)	0.841
SBP (mmHg)	124.00 (122.55, 130.13)	121.00 (120.73, 130.45)	−1.20 (−5.02, 2.61)	0.657
DBP (mmHg)	85.00 (81.50, 87.72)	80.00 (78.36, 85.91)	−3.16 (−5.73, −0.59)	0.087
**Glycometabolism indices**				
Fasting glucose (mmol/L)	5.35 (5.17, 5.95)	5.19 (4.94, 5.45)	−0.36 (−0.72, −0.01)	0.034^*^
HOMA-IR	3.63 ± 2.06	2.77 ± 1.35	−0.84 (−1.55, −0.13)	0.009^*^
**Lipid metabolism indices**				
TC (mmol/L)	5.09 ± 0.99	4.48 ± 0.76	−0.61 (−0.86, −0.36)	<0.001^**^
TG (mmol/L)	1.80 (1.72, 2.81)	1.89 (1.62, 2.11)	−0.40 (−0.87, 0.07)	0.232
HDL-C (mmol/L)	1.08 ± 0.23	1.05 ± 0.18	−0.03 (−0.09, 0.04)	0.432
LDL-C (mmol/L)	3.33 ± 0.83	2.95 ± 0.69	−0.37 (−0.60, −0.15)	0.002^*^
Apo A (g/L)	1.09 (1.04, 1.14)	1.06 (1.05, 1.18)	0.03 (−0.04, 0.09)	0.53
Apo B (g/L)	0.91 ± 0.18	0.77 ± 0.16	−0.13 (−0.18, −0.08)	<0.001^**^
**Sleep data**				
AHI	53.95 (49.65, 61.92)	20.35 (18.74, 30.31)	−31.26 (−37.16, −25.35)	<0.001^**^
Mean SaO_2_ (%)	92.20 (90.96, 93.08)	95.00 (94.15, 95.33)	2.72 (1.73, 3.71)	<0.001^**^
LSpO_2_ (%)	73.47 ± 9.77	82.34 ± 8.34	8.87 (5.82, 11.92)	<0.001^**^
CT90 (%)	21.78 (18.29, 30.06)	2.44 (3.63, 11.23)	−16.75 (−21.93, −11.57)	<0.001^**^
ODI	55.26 ± 19.63	27.50 ± 20.33	−27.77 (−33.92, −21.61)	<0.001^**^
MAI	40.03 ± 26.70	29.05 ± 17.67	−10.98 (−18.98, −2.99)	0.008^*^
ESS	10.30 ± 5.61	6.59 ± 5.06	−3.45 (−5.10, −1.81)	<0.001^**^

Abbreviations: BMI = body mass index, WC = waist circumference, SBP = systolic blood pressure, DBP = diastolic blood pressure, HOMA-IR = homeostasis model of assessment for insulin resistance index, TC = total cholesterol, TG = total triglycerides, HDL-C = high-density lipoprotein cholesterol, LDL-C = low-density lipoprotein cholesterol, Apo A = apolipoprotein A, Apo B = apolipoprotein B, AHI = apnea-hypopnea index, SaO_2_ = oxygen saturation, LSpO_2_ = lowest pulse oxygen saturation, CT90 = percentage of time during which oxyhemoglobin saturation was < 90%, ODI = oxygen desaturation index, MAI = micro-arousal index, ESS = Epworth Sleepiness Scale, CI = confidence interval.

*p < 0.05; **p < 0.001.

Neither BMI nor WC of the patients changed significantly after surgery. Table 2 lists the changes in blood pressure indices, glycometabolism indices, and lipid metabolism indices. Reductions were evident in a number of cardiovascular-related biomarkers, including fasting glucose (p < 0.05), HOMA-IR (p < 0.05), fasting TC (p < 0.001), fasting LDL-C (p < 0.05), and fasting Apo B (p < 0.001). SBP, DBP, TG, HDL-C, and Apo A did not change significantly after surgery.

### Changes in ten endothelial function markers

Table 3 lists the preoperative and postoperative serum levels of endothelial function-related markers. Reductions of serum levels of VEGFA, Ang2, E-selectin, VWF, VWFCP, and TM after surgery were significant (P < 0.05). Levels of Ang1 and ICAM1 increased without significance, and levels of VEGFR1 and VCAM decreased without significance. We also searched for normal values of 10 endothelium function-related markers in previous published studies. Preoperative and postoperative serum levels of VEGFA. VEGFR1, VCAM, E-selectin and TM were lower than healthy values. Preoperative and postoperative serum levels of Ang1, Ang2, ICAM1, VWF and VWFCP were higher than healthy values. Preoperative level of VEGFR1 in male subgroup was higher than female subgroup. Compared with male subgroup, the preoperative serum level of E-selectin in female subgroup was higher. There was no significant difference in preoperative level of other endothelial cytokines between different sex. We also found no evident difference of postoperative serum values of 10 EC-function related markers between male and female subgroups.

**Table 3:** Comparisons of serum levels of ten endothelial function-related markers between preoperative and postoperative and between male and female.

Variables	Preoperative	Postoperative						*P value*	Mean change (95%CI)	*P value*	Health
		Male	Female	*P value*		Male	Female				
VEGFA (pg/ml)	40.29 (37.94, 70.29)	43.67(39.24,65.55)	17.98(−33.10,159.47)	0.377	23.34 (25.94, 41.70)	20.19(25.52,43.22)	25.75(8.76,53.08)	0.875	−20.29 (−35.27, −5.31)	0.013^*^	69.8 ± 11^54^
VEGFR1 (pg/ml)	1160.96 (1054.77, 1563.63)	1.18(1.11,1.68)	0.60(0.25,1.44)	0.027*	1079.38 (1100.76, 1632.89)	1.28(1.18,1.77)	0.77(0.26,1.30)	0.062	57.63 (−120.85, 236.10)	0.852	1892 ± 1200^55^
Ang1 (ng/ml)	6.84 (6.22, 7.46)	6.72(6.18,7.3)	6.87(4.10,10.63)	0.95	7.64 (6.81, 8.48)	6.87(6.71,8.62)	6.35(5.50,9.54)	0.975	0.81 (−0.01, 1.61)	0.07	3 ± 0.432^56^
Ang2 (ng/ml)	2.96 (2.87, 3.05)	2.95(2.86,3.07)	2.97(2.71,3.08)	0.637	2.89 (2.80, 2.98)	2.87(2.80,3.00)	2.91(2.66,3.06)	0.9	−0.06 (−0.16, 0.03)	0.042^*^	0.88 ± 0.4^57^
ICAM1 (ng/ml)	1215.95 ± 380.88	0.37(0.35,0.43)	0.34(0.28,0.41)	0.489	1271.36 ± 358.78	0.33(0.31,0.39)	0.31(0.23,0.44)	0.73	55.41 (−83.66, 194.49)	0.426	312.58 ± 79.77^58^
VCAM (ng/ml)	188.53 (209.66, 346.77)	196.24(202.76,307.03)	167.37(−9.80,802.16)	0.925	176.02 (196.18, 266.89)	175.06(190.97,267.79)	176.98(121.95,363.88)	0.73	−46.68 (−108.63, 15.27)	0.176	975.35 ± 389.32^58^
E-selectin (ng/ml)	38.80 ± 17.99	32.43(30.61,42.62)	47.22(37.67,63.09)	0.035*	31.59 ± 15.21	27.20(25.36,35.56)	32.43(24.31,50.80)	0.228	−7.21 (−11.01, −3.41)	<0.001^**^	58.67 ± 24.09^58^
VWF (ng/ml)	289.44 (251.93, 326.94)	302.77(237.90,323.36)	304.48(276.27,385.15)	0.45	230.61 (190.73, 270.48)	207.13(193.76,282.98)	130.31(81.91,297.23)	0.377	−58.83 (−103.93, −13.73)	0.016^*^	134.65 ± 15.54^58^
VWFCP (ng/ml)	381.35 ± 115.74	375.73(348.12,429.30)	342.91(277.06,407.88)	0.489	347.83 ± 112.93	327.90(312.20,388.60)	307.99(231.34,437.22)	0.73	−33.52 (−66.34, −0.70)	0.046^*^	79.47 ± 10.78^59^
TM (ng/ml)	0.28 ± 0.12	0.27(0.24,0.31)	0.24(0.15,0.40)	0.778	0.22 ± 0.07	0.20(0.20,0.25)	0.20(0.17,0.24)	0.95	−0.06 (−0.09, −0.03)	0.001*	32.0 ± 67.6^60^

Abbreviations: VEGFA = vascular endothelium growth factor A, VEGFR1 = vascular endothelium growth factor receptor 1, Ang1 = angiopoietin-1, Ang2 = angiopoietin-2, ICAM1 = intercellular adhesion molecule-1, VCAM = vascular cell adhesion molecule, E-selectin = endothelial-leukocyte adhesion molecule, VWF = von willebrand factor, VWFCP = von willebrand factor cleavage protease, TM = thrombomodulin, CI = confidence interval.

*p < 0.05.

Table 4 presents subgroup comparisons. VEGFA was associated with preoperative Apo A level (p < 0.05 between normal and abnormal subgroups); VEGFR1 was associated with preoperative hypertension (p < 0.05); VWF was associated with surgery success (p < 0.05); and TM was associated with preoperative WC (p < 0.05 for abdominal obesity), follow-up duration (p < 0.05 between <1 year and >1 year subgroups), and preoperative hypertension (p < 0.05).

**Table 4:** Subgroup comparisons of 10 serum markers with the baseline values.

	Mean change of Ang1 (95%CI)	Mean change of Ang2 (95%CI)	Mean change of VEGFA (95%CI)	Mean change of VEGFR1 (95%CI)	Mean change of vWF (95%CI)	Mean change of vWFCP (95%CI)	Mean change of TM (95%CI)	Mean change of ICAM1 (95%CI)	Mean change of VCAM (95%CI)	Mean change of E-selectin (95%CI)
**Sex**										
Female	0.93 (−0.01, 1.87)	−0.07 (−0.17, 0.03)	−18.03 (−31.75, −4.03)	80.03 (−131.57, 291.64)	−43.26 (−91.84, 5.31)	−38.31 (−75.39, −1.23)	−0.05 (−0.09, −0.02)	49.16 (−92.32, 190.64)	−26.51 (−65.89, 12.86)	−6.15 (−10.43, −1.88)
Male	0.15 (−1.29, 1.60)	−0.04 (−0.38, 0.30)	−32.26 (−109.82, 45.30)	−60.82 (−230.39, 108.76)	−141.13 (−275.05, −7.22)	−8.19 (−93.04, 76.66)	−0.06 (−0.17, 0.05)	88.45 (−509.37, 686.28)	−153.27 (−564.90, 258.36)	−12.83 (−21.60, −4.06)
**AHI**										
<30/h	0.67 (−1.55, 2.89)	0.16 (−0.53, 0.84)	−3.70 (−23.47, 16.06)	−140.30 (−413.44, 132.84)	60.47 (−506.95, 627.89)	47.38 (−4.60, 99.37)	−0.09 (−0.41, 0.22)	−230.65 (−1166.52, 705.22)	−37.44 (−302.28, 227.40)	−2.26 (−36.55, 32.02)
≥30/h	0.82 (−0.05, 1.68)	−0.08 (−0.18, 0.02)	−21.51 (−37.56, −5.46)	72.11 (−119.03, 263.25)	−67.56 (−112.09, −23.03)	−39.44 (−73.98, −4.89)	−0.05 (−0.08, −0.02)	76.34 (−68.74, 221.42)	−47.36 (−113.61, 18.90)	−7.58 (−11.52, −3.63)
**BMI**										
<28 kg/m^2^	0.87 (−0.42, 2.16)	−0.06 (−0.21, 0.09)	−15.83 (−31.89, 0.23)	82.12 (−152.94, 317.18)	−32.12 (−88.57, 24.32)	−38.96 (−76.51, −1.41)	−0.06 (−0.10, −0.03)	67.59 (−103.12, 238.30)	−30.05 (−80.06, 19.96)	−6.47 (−10.77, −2.16)
≥28 kg/m^2^	0.37 (−0.16, 1.50)	−0.05 (−0.15, 0.04)	−19.42 (−35.29, −3.54)	47.71 (−138.89, 324.31)	−64.79 (−111.20, −18.38)	−35.08 (−69.51, −0.65)	−0.05 (−0.08, −0.02)	29.74 (−116.01, 175.48)	−50.30 (−116.79, 16.19)	−7.91 (−11.91, −3.91)
**Abdominal obesity**										
No	2.64 (−4.01, 9.28)	−0.20 (−1.36, 0.96)	−32.29 (−124.62, 60.05)	193.13 (−1357.47,1743.74)	22.59 (−408.39, 453.56)	−12.14 (−282.56, 258.28)	−0.17 (−0.30, −0.03)*	406.32 (−68.78, 881.42)	2.80 (−82.44, 88.04)	2.27 (−0.43, 4.97)
Yes	0.37 (−0.16, 1.50)	−0.05 (−0.15, 0.04)	−19.42 (−35.29, −3.54)	47.71 (−138.89, 324.31)	−64.79 (−111.20, −18.38)	−35.08 (−69.51, −0.65)	−0.05 (−0.08, −0.02)*	29.74 (−116.01, 175.48)	−50.30(−116.79, 16.19)	−7.91(−11.91, −3.91)
**Follow-up time**										
<1 year	1.04 (−0.56, 2.63)	−0.17 (−0.41, 0.07)	−13.04 (−26.53, 0.44)	133.61 (−256.04, 523.25)	−85.46 (−154.03, −16.89)	−49.25 (−126.88, −28.37)	−0.01 (−0.04, 0.03)	−17.08 (−276.17, 242.01)	6.07 (−95.45, 107.59)	−8.17 (−14.59, −1.74)
≥1 year	0.72 (−2.25, 1.71)	−0.03 (−1.33, 0.08)	−22.71 (−42.49, −2.93)	32.30 (−179.06, 243.66)	−49.96 (−107.112, 7.21)	−28.27 (−66.08, 9.54)	−0.07 (−0.11, −0.03)*	79.58 (−91.32, 250.48)	−64.26 (−141.44, 12.92)	−6.90 (−11.67, −2.12)
**Surgery**										
Unseccessful	0.94 (−0.58, 2.45)	−0.02 (−0.15, 0.11)	−20.58 (−41.57, 0.42)	176.66 (−111.68, 465.00)	−14.07 (−81.97, 53.84)*	−25.71 (−72.75, 21.33)	−0.07 (−0.11, −0.03)	37.72 (−182.34, 257.79)	−53.27 (−104.23, −2.30)	−9.14 (−15.55, −2.72)
Successgul	0.68 (−0.05, 1.41)	−0.11 (−0.26, 0.04)	−20.01 (−43.17, 3.15)	−61.41 (−284.44, 161.62)	−103.60 (−162.09, −45.11)*	−41.33 (−90.85, 8.20)	−0.04 (−0.86, 0.01)	73.10 (−115.77, 261.97)	−40.09 (−158.83, 78.64)	−5.29 (−9.79, −0.80)
**Preoperative hypertention**										
No	0.65 (−0.37, 1.66)*	−0.02 (−0.12, 0.09)	−25.27 (−44.87, −5.67)	53.72 (−168.50, 275.94)	−45.18 (−103.00, 12.65)	−36.00 (−72.80, 0.81)	−0.07 (−0.11, −0.03)*	75.91 (−90.67, 242.49)	−47.07 (−127.37, 33.23)	−7.03 (−11.91, −2.15)
Yes	1.29 (−0.04, 2.61)*	−0.21 (−0.45, 0.03)	−5.36 (−17.71, 7.00)	69.33 (−249.23, 387.89)	−99.80 (−156.27, −43.34)	−26.08 (−109.82, 57.67)	−0.01 (−0.06, 0.04)*	−6.08 (−297.35, −285.18)	−45.51 (−126.16, 35.15)	−7.78 (−13.21, −2.34)
**Preoperative hyperglycaemia**										
No	0.94 (0.05,1.83)	−0.04 (−0.14, 0.05)	−21.99 (−38.78, −5.20)	118.23 (−69.42, 305.88)	−68.31 (−116.13, −20.48)	−35.38 (−71.29, 0.53)	−0.06 (−0.09, −0.02)	96.49 (−52.10, 245.08)	−51.94 (−121.55, 17.67)	−5.79 (−8.94, −2.65)
Yes	−0.25 (−2.20, 1.70)	−0.22 (−0.79, 0.34)	−7.06 (−30.95, 16.83)	−415.12 (−978.80, 148.56)	15.07 (169.47, 199.61)	−18.97 (−132.65, 94.71)	−0.05 (−0.15, 0.05)	−265.02 (−674.28, −144.25)	−5.69 (−101.03, 89.66)	−18.28 (−49.00, 12.44)
**Preoperative IR**										
No	1.19 (−0.59, 2.97)	0.04 (−0.14, 0.22)	−20.60 (−46.90, 5.69)	114.43 (−101.04, 329.91)	−59.63 (−148.06, 28.80)	−21.85 (−58.14, 14.43)	−0.06 (−0.11, 0.01)	66.04 (−135.33, 267.41)	24.59 (−55.13, 104.31)	−9.80 (−19.60, −0.01)
Yes	0.61 (−0.28, 1.50)	−0.12(−0.23, −0.01)	−20.13(−39.53, −0.73)	28.24(−227.19, 283.67)	−58.42(−113.60, −3.24)	−39.55(−87.12, 8.01)	−0.05 (−0.09, −0.02)	49.91(−142.22, 242.05)	−83.55(−167.98, 0.89)	−0.87 (−9.23, −2.51)
**Preoperative level of TC**										
Normal	0.31 (−0.51, 1.13)	−0.06 (−0.16, 0.03)	−21.42 (−47.43, 4.60)	108.41 (−200.30, 417.11)	−89.14 (162.12,16.16)	−33.03 (−81.25, 15.19)	−0.05 (−0.10, 0.01)	2.41 (−212.86, 217.68)	−66.08(−185.80,53.63)	−9.81 (−16.08, −3.54)
Borderline	1.66 (−0.31, 3.62)	−0.04 (−0.26, 0.18)	−21.62 (−45.00, 1.76)	128.73 (−141.48, 398.94)	−68.57 (−121.74, −15.40)	−51.36 (−97.49, −5.23)	−0.06 (−0.10, −0.01)	28.29 (−202.42, 259.00)	−2.05 (−46.27, 42.17)	−6.67 (−12.79, −0.56)
Abnormal	0.55 (−1.75, 2.86)	−0.12 (−0.52, 0.28)	−13.92 (−45.07, 17.22)	−254.35 (−519.01, 10.31)	57.29 (−80.02, 194.60)	3.18 (−138.73, 145.09)	−0.08 (−0.16, 0.01)	280.10 (−132.04, 692.24)	−81.34 (−211.28, 48.61)	−0.21 (−6.97, 6.55)
**Preoperative level of TG**										
Normal	0.88 (−0.08, 1.85)	−0.06 (−0.14, 0.02)	−16.26 (−32.01, −0.52)	165.57 (−133.70, 464.84)	−69.87 (−136.32, −3.41)	−25.00 (−79.50, 29.50)	−0.04 (−0.09, 0.01)	−67.92 (−283.50, 147.67)	−31.34 (−97.22, 34.54)	−6.93 (−11.08, −2.79)
Borderline	1.34 (−1.36, 4.05)	−0.06 (−0.36, 0.24)	−21.84 (−65.76, 22.09)	−4.85 (−303.07, 293.36)	−82.85 (−179.75, 14.04)	−44.50 (−136.34, 47.35)	−0.04 (−0.11, 0.04)	340.48 (29.60, 651.36)	−14.61(−121.45, 92.24)	−3.90(−10.17, 2.37)
Abnormal	0.31(−1.17, 1.80)	−0.07(−0.30, 0.17)	−24.95 (−60.15, 10.25)	−51.96(−411.66, 307.75)	−25.91(−123.93, 72.11)	−37.84(−86.44, 10.76)	−0.09 (−0.14, −0.03)	27.97(−206.25, 262.19)	−91.51(−264.35, 81.33)	−9.98(−20.51, 0.54)
**Preoperative level of HDL-C**										
Lower	0.60(−0.33, 1.53)	−0.10(−0.18, −0.01)	−21.37(−46.71, 3.96)	57.63(−258.07, 373.34)	−79.19(−135.64, −22.73)	−36.49(−88.73, 15.74)	−0.04(−0.08, 0.01)	5.49(−222.54, 233.52)	−58.52(−181.91, 64.88)	−8.08(−15.26, −0.90)
Normal	0.98(−0.35, 2.30)	−0.04(−0.20, 0.13)	−19.40(−38.82, 0.03)	57.62(−161.14, 276.38)	−41.87(−113.11, 29.37)	−31.04(−76.28, 14.21)	−0.07(−0.11, −0.03)	97.01(−87.60, 281.62)	−36.82(−96.50, 22.86)	−6.49(−10.64, −2.34)
**Preoperative level of LDL-C**										
Normal	0.74(−0.39, 1.87)	−0.06(−0.20, 0.08)	−13.28 (−26.66, 0.10)	32.47(−203.78, 268.73)	−55.03(−115.72, 5.66)	−32.05(−81.47, 17.38)	−0.05 (−0.09, −0.02)	95.18(−54.91, 245.28)	−14.89(−68.51, 38.73)	−5.93(−10.58, −1.28)
Borderline	0.08(−0.68, 0.84)	−0.05(−0.16, 0.06)	−32.86(−70.59, 4.82)	9.44(−378.45, 397.32)	−78.44(−182.59, 25.71)	−43.58(−111.29, 24.12)	−0.05(−0.13, 0.02)	−51.02(−409.31, 307.28)	−102.87(−289.92, 84.18)	−11.36(−20.68, −2.03)
Abnormal	2.47(−1.34, 6.29)	−0.10(−0.55, 0.35)	−18.14 (−77.26, 40.97)	236.64 (−312.27, 785.56)	−32.12(−138.43, 74.19)	−18.21(−85.52, 49.09)	−0.07(−0.17, 0.04)	137.59 (−258.28, 533.46)	−38.75(−84.79, 7.29)	−3.15(−10.62, 4.32)
**Preoperative level of Apo A**										
Normal	1.62(−0.61, 3.85)	0.15(−0.10, 0.40)*	−26.70 (−57.67, 4.26)	−21.25(−459.17, 416.67)	−27.68(−133.92, 78.56)	−5.37(−87.30, 76.57)	−0.06(−0.14, 0.01)	161.40 (−190.65, 513.45)	−30.67(−169.99, 108.66)	−5.25(−12.45, 1.95)
Abnormal	0.50(−0.31, 1.31)	−0.14(−0.23, −0.06)*	−17.89 (−35.93, 0.15)	87.20(−112.29, 286.69)	−70.52(−121.61, −19.42)	−44.07 (−79.84, −8.31)	−0.05(−0.09, −0.02)	15.67 (−135.30, −166.64)	−52.68(−125.14, 19.78)	−7.95(−12.64, −3.26)
**Preoperative level of Apo B**										
Lower	0.39(−0.71, 1.48)	−0.04(−0.18, 0.10)	−26.36 (−60.96, 8.25)	46.67(−301.04, 394.38)	−83.92(−170.22, 2.39)	−37.70(−99.01, 23.61)	−0.03(−0.10, 0.04)	−42.04(−307.80, 223.71)	−83.14(−261.28, 95.01)	−8.35(−13.10, −3.60)
Normal	0.93(−0.16, 2.03)	−0.09(−0.20, 0.02)	−13.09(−29.74, 3.56)	128.26(−166.16, 422.69)	−68.92(−133.56, −4.28)	−37.44(−85.25, 10.36)	−0.06 (−0.09, −0.02)	45.04(−166.67, 256.75)	0.82(−38.20, 39.83)	−9.08(−16.18, −1.97)
Higher	1.25(−2.30, 4.81)	−0.05(−0.51, 0.42)	−27.83 (−73.50, 17.83)	−107.25 (−353.67, 139.16)	14.67(−106.95, 136.29)	−15.37(−118.05, 87.32)	−0.10 (−0.18, −0.01)	265.38(−42.63, 573.38)	−103.00 (−201.41, −4.59)	−0.19 (−5.97, 5.59)

Abbreviations: AHI = apnea-hypopnea index, BMI = body mass index, IR = insulin resistance, SBP = systolic blood pressure, DBP = diastolic blood pressure, HOMA-IR = homeostasis model of assessment for insulin resistance index, TC = total cholesterol, TG = triglycerides, HDL-C = high-density lipoprotein cholesterol, LDL-C = low-density lipoprotein cholesterol, Apo A = apolipoprotein A, Apo B = apolipoprotein B, Ang1 = angiopoietin-1, Ang2 = angiopoietin-2, VWF = von willebrand factor, VWFCP = von willebrand factor cleavage protease, TM = thrombomodulin, E-selectin = endothelial-leukocyte adhesion molecule, ICAM1 = intercellular adhesion molecule-1, VCAM = vascular cell adhesion molecule, VEGFA = vascular endothelium growth factor A, VEGFR1 = vascular endothelium growth factor receptor 1, CI = confidence interval.

*p < 0.05.

Table 5 shows that among the biomarkers that improved significantly after surgery, Spearman’s correlation test revealed positive associations between ΔAng2 and ΔApo B (r = 0.372, p = 0.013), ΔVEGFA and ΔCT90% (r = 0.298, p = 0.049), ΔVWFCP and Δglucose (r = 0.317, p = 0.036), and ΔTM and ΔCT90% (r = 0.343, p = 0.023).

**Table 5:** Spearman’s correlations between changes in serum levels of 10 markers, PSG parameters and biomarker levels.

	ΔAHI	ΔMean SaO_2_	ΔLSpO_2_	ΔCT90%	ΔODI	ΔMAI	ΔESS	ΔGlucose	ΔHOMA-IR	ΔTC	ΔLDL-C	ΔApoB
ΔVEGFA	0.242	−0.178	−0.054	0.298^*^	0.259	−0.069	−0.089	0.051	0.137	−0.083	−0.068	−0.122
ΔAng2	−0.11	0.157	−0.058	−0.2	0.161	−0.009	0.061	0.07	−0.025	0.121	0.236	0.372^*^
ΔE-selectin	−0.227	0.11	−0.093	−0.135	−0.061	0.113	0.111	0.1	−0.166	0.187	0.146	0.18
ΔvWF	0.004	0.074	−0.07	−0.28	−0.043	−0.151	0.027	0.012	−0.268	−0.12	−0.032	−0.173
ΔvWFCP	−0.012	0.112	−0.086	−0.075	0.244	0.021	0.106	0.317^*^	0.088	0.066	0.014	0.237
ΔTM	0.191	−0.211	0.066	0.343^*^	0.139	0.202	−0.061	−0.07	−0.056	0.064	0.084	0.022

Abbreviations: VEGFA = vascular endothelium growth factor A, Ang2 = angiopoietin-2, E-selectin = endothelial-leukocyte adhesion molecule, vWF = von willebrand factor, vWFCP = von willebrand factor cleavage protease, TM = thrombomodulin, AHI = apnea-hypopnea index, SaO_2_ = oxygen saturation, LSpO_2_ = lowest pulse oxygen saturation, CT90 = percentage of time during which oxyhemoglobin saturation was < 90%, ODI = oxygen desaturation index, MAI = micro-arousal index, ESS = Epworth Sleepiness Scale, HOMA-IR = homeostasis model of assessment for insulin resistance index, TC = total cholesterol, LDL-C = low-density lipoprotein cholesterol, Apo B = apolipoprotein B.

*p < 0.05.

In Table 6, multivariable linear regression analyses showed that VEGFA levels were significantly associated with CT90% (βcoefficient = 1.071, p = 0.009) and glucose (βcoefficient = 13.406, p = 0.021). Ang2 levels were significantly associated with CT90% (βcoefficient = 0.007, p = 0.008), ODI (βcoefficient = 0.008, p = 0.001), Glucose (coefficient = −0.069, p = 0.048) and Apo B (βcoefficient = 0.896, p = 0.001).

**Table 6:** Multivariable linear regression analyses with changes in serum levels of 10 markers, PSG parameters and biomarker levels.

	ΔAHI	ΔMean SaO_2_	ΔLSpO_2_	ΔCT90%	ΔODI	ΔMAI	ΔESS	ΔGlucose	ΔHOMA-IR	ΔTC	ΔLDL-C	ΔApoB
ΔVEGFA	0.001	6.334	0.423	1.071*	0.142	−0.131	−0.817	13.406*	−1.859	9.299	−25	−52.019
ΔAng2	−0.004	−0.009	−0.007	−0.007*	0.008*	−0.003	0.014	−0.069*	0.009	−0.013	0.033	0.896*
ΔE-selectin	0.02	0.23	−0.41	−2.649	5.112	−0.008	0.319	39.159	−9.695	−0.886	−4.8	101.277
ΔvWF	1.073	−11.994	−3.123	−5.123	0.399	−0.669	2.951	−9.962	−9.847	45.051	−4.8	−260.24
ΔvWFCP	−4.076	−8.073	−0.41	−2.649	5.112	−0.008	0.319	39.159	−9.695	−0.886	−30.332	101.277
ΔTM	0.001	0.01	0.003	0.005	−0.001	0	−0.001	−0.005	−0.006	0.038	−0.001	−0.087

Abbreviations: VEGFA = vascular endothelium growth factor A, Ang2 = angiopoietin-2, E-selectin = endothelial-leukocyte adhesion molecule, vWF = von willebrand factor, vWFCP = von willebrand factor cleavage protease, TM = thrombomodulin, AHI = apnea-hypopnea index, SaO_2_ = oxygen saturation, LSpO_2_ = lowest pulse oxygen saturation, CT90 = percentage of time during which oxyhemoglobin saturation was < 90%, ODI = oxygen desaturation index, MAI = micro-arousal index, ESS = Epworth Sleepiness Scale, HOMA-IR = homeostasis model of assessment for insulin resistance index, TC = total cholesterol, LDL-C = low-density lipoprotein cholesterol, Apo B = apolipoprotein B.

*p < 0.05.

## Discussion

Our pilot study might be the first to explore changes of 10 risk markers of endothelial function in patients with OSA after upper-airway surgery. In comparison with previous research, we found similar declining tendency in ICAM1^7^, E-selectin^8^ and VWF^10^ and increasing tendency in VCAM^6^. We found that upper-airway surgery can lead to significant improvements in the levels of endothelial function-related markers, including VEGFA, Ang2, E-selectin, VWF, VWFCP, and TM. We also established correlations between VEGFA, TM, VWFCP, and Ang2 and ameliorated oxyhemoglobin saturation and glucolipid metabolism.

Several prior studies have focused on the pernicious impact of OSA-related pathological features on vascular endothelial dysfunction, such as increases in EC instability molecules^14,15^, circulating cell adhesion cytokines^6,26^, and hyper-coagulability^27,28^. Recurrent intermittent hypoxia during sleep and the concomitant high level of endothelial nitric oxide^29,30^ aggravate oxidative stress^31^ and systemic inflammation^32^. Endothelial dysfunction is considered to be one of the crucial mechanisms leading to adverse cardiovascular consequences in OSA patients^33^.

In our study, we were pleased to find that the reductions in levels of VEGFA and Ang2 were significant. Therefore, the synergistic effects of VEGFA^34–36^ and Ang2^37–40^ on EC instability and hyperpermeability were significantly reduced by upper-airway surgery. Higher expressions of VEGFR1 and Ang1, as representational markers of protection and stabilization, were increased after upper-airway surgery, although the changes were not statistically significant. Together with the increased Ang1/Ang2^40^ ratio, this finding implies a reduced risk of CVD by reducing EC permeability and promoting healthy status of vessels after surgery. Hypoxia, even hypoxia-induced oxidative stress, coagulation, inflammation, and/or a hyperlipidemic environment, can all induce release of CAMs^8,41–43^. Postoperative results indicated that surgical treatment decreased expression of VCAM and E-selectin^44^, (regardless of whether upper-airway surgery outcome was successful), along with evident improvement of sleep parameters. However, the serum level of ICAM1 was increased. This finding suggests that initial CAM-induced risk of atherosclerosis formation still existed, and other therapies are necessary to eliminate the possibility of atherosclerosis progression. In our study, we observed decreases in VWF and TM accompanied with reduction of VWFCP (all significant). Reduction in VWFCP^44,45^ may be due to synchronization with low VWF, so this finding implies that long-term risk of CVD, particularly the possibility of artery atherosclerosis and thrombus, might be significantly reduced after surgical therapy. Almost all of the ten endothelial function markers were improved to some extent postoperatively, with the exception of ICAM1. We found that most of the values in post-operative subjects were higher than healthy individuals (normal values of previous published studies as reference). Upper-airway surgery might not reverse values of endothelial function in a relative short follow-up time. Gender seemed not to be associated with these endothelial cytokines we focused on.

We also found that different preoperative conditions (including sex, AHI, BMI, fasting glucose levels, fasting insulin levels, fasting TC levels, fasting TG levels, fasting HDL-C levels, fasting LDL-C levels, and fasting Apo B levels) might not be associated with the mean change of the ten endothelial function markers. Only respective markers in respective conditions, i.e., VEGFA (with or without preoperative hypertension), VEGFR1 (with or without normal level of fasting Apo A), VWF (successful or unsuccessful surgery), TM (with or without preoperative abdominal obesity, follow-up time >1 year or not, and with or without preoperative hypertension), differed significantly, with similar variation tendencies. This finding implies that the beneficial incomes of improved endothelial function after surgery were relatively steady.

After comprehensively comparing the changes of sleep parameters, glucolipid metabolism indices, and endothelial function-related markers, we found improvements of the potent markers VEGFA and TM might be associated with down-regulation of CT90%. Improvement of Ang2 was associated with down-regulation of fasting Apo B levels. Improvement of VWFCP might be associated with down-regulation of fasting glucose levels. Improvement of VEGFA could be associated with melioration of CT90% and fasting glucose levels, especially with down-regulation of blood glucose level. Improvement of Ang2 was associated with down-regulation of CT90%, ODI, fasting glucose levels and fasting Apo B levels, in especial with melioration of Apo B levels. These findings suggest that improved endothelial function-related markers are associated with ameliorated oxyhemoglobin saturation and glucolipid metabolism indices after upper-airway surgery.

Our findings are consistent with results of experimental studies reporting vascular hyperpermeability, hypercoagulability, and unbalance of the steady endovascular environment in response to chronic intermittent hypoxia^46,47^ and impaired sleep architecture^48^. Potential physiological pathways may include oxidative stress, inflammation, and sympathetic activation^49–51^.

Several limitations to this study should be acknowledged. First, the sample size in our pilot study was small, so selection bias might exist. Second, this study was not a randomized controlled trial (RCT); although a RCT could upgrade the level of evidence and identify the causal relationships, it is difficult to perform one in this context. Third, confounding factors, such as diet and exercise, could also affect endothelial function markers. Fourth, we did not measure endothelial dysfunction assessed by flow-mediated dilatation (FMD). Fourth, though we search for normal values of 10 endothelium function-related markers in previous published studies, we did not set a control group simultaneously. Finally, this was a preliminary hypothesis-generating study; we plan to confirm the results in an independent sample of patients in the near future. Further more, given that the majority of endothelium function-related markers influence the process of endothelial colony-forming cells (ECFC)^52,53^ transforming into mature EC, we consider that further research about isolated ECFC from circulation is essential to explore the potential pathological procedure of endothelial injury induced by unbalanced EC markers. We suspect that intermittent hypoxia and sleep fragmentation is likely the key factors.

In conclusion, the results of this study suggest that upper-airway surgery might be associated with an improvement in endothelial dysfunction-related markers in patients with OSA, probably through downregulation of inflammatory adhesion molecules, endothelial cell (EC) propermeability, inflammatory factor overflow, and hypercoagulability and upregulation of endothelium-stabilizing markers. The improvements in endothelial function biomarkers might be related to increased oxygen saturation after surgery. More prospective research on this issue will be needed to verify our findings and demonstrate the potential benefit of airway surgery for OSA patients Fig. 1.

## Data Availability

The corresponding authors will provide the accessibility of clinical data applied to support conclusions after receiving request.
